# MicroRNA in prostate cancer: functional importance and potential as circulating biomarkers

**DOI:** 10.1186/1471-2407-14-930

**Published:** 2014-12-10

**Authors:** Benjamin L Jackson, Anna Grabowska, Hari L Ratan

**Affiliations:** Unit of Cancer Biology, University of Nottingham, Queens Medical Centre, Derby Road, Nottingham, NG7 2UH England; Department of Urology, Nottingham City Hospital, Hucknall Road, Nottingham, NG5 1PB England

**Keywords:** Prostatic neoplasms, MicroRNAs, Biological markers

## Abstract

**Background:**

This non-systematic review article aims to summarise the progress made in understanding the functional consequences of microRNA (miRNA) dysregulation in prostate cancer development, and the identification of potential miRNA targets as serum biomarkers for diagnosis or disease stratification.

**Results:**

A number of miRNAs have been shown to influence key cellular processes involved in prostate tumourigenesis, including apoptosis-avoidance, cell proliferation and migration and the androgen signalling pathway. An overlapping group of miRNAs have shown differential expression in the serum of patients with prostate cancer of varying stages compared with unaffected individuals. The majority of studies thus far however, involve small numbers of patients and have shown variable and occasionally conflicting results

**Conclusion:**

MiRNAs show promise as potential circulating biomarkers in prostate cancer, but larger prospective studies are required to validate particular targets and better define their clinical utility.

## Review

Despite incremental advances, there remain a number of challenges yet to be overcome in the management of prostate cancer. The increasing use of PSA-based screening has facilitated the early detection of prostate cancer at a curable stage of the disease, however the problem of determining which of these patients truly require treatment and which can be safely observed remains. The use of nomograms based on clinical parameters is helpful, but the search for additional biomarkers to “fine-tune” risk stratification continues.

MicroRNAs (miRNAs) are short (18-22 nucleotide) non-coding RNA sequences. They were first identified in nematodes in 1993 by Lee *et al.*[1], and, to date, over 1000 separate miRNA sequences have been identified in the human [2]. It is becoming clear that miRNAs represent a vast, previously unrecognised layer of molecular signalling in eukaryotes, and that miRNAs play an important role in the regulation of protein expression [3].

MiRNAs present an appealing target for biomarker discovery. Each miRNA possesses the ability to interact with a number of cellular pathways, and consequently changes in the expression of a small number of miRNAs may reflect dysregulation of a broad range of cellular processes, in keeping with the complexity of neoplasia. Furthermore, miRNAs are relatively resistant to RNase degradation due to their short sequence length which improves their longevity in both serum and tissue samples [4]. A number of techniques for miRNA profiling have been developed [5], and the principle that increased tissue expression of miRNAs may lead to elevated serum levels has been demonstrated experimentally [6].

It may eventually be possible to identify a panel of miRNAs that can be measured in serum or tissue and used to better establish which patients with localised prostate cancer will ultimately progress and should therefore be treated aggressively, or indeed to establish which patients with more advanced disease are most likely to benefit from particular therapies, including novel agents acting on the androgen pathway. Serum miRNA profiling is especially exciting given that it is accessible in a non-invasive manner, and it may be possible to establish a diagnostic serum miRNA panel that would reduce the need for prostate biopsies in patients with elevated PSA levels, some of whom ultimately will not have prostate cancer.

None of these aims have yet been met, but this review aims to summarise the progress made both with understanding the role of miRNA dysregulation in prostate cancer tumourigenesis, and with establishing which miRNAs may be useful serum biomarkers.

### Functional role of miRNA dysregulation in prostate cancer

The cellular characteristics required for the initiation and progression of cancer have been described and involve a broad set of biological pathways including those that drive cell growth, inhibit cell death, and promote angiogenesis and metastasis [7]. Given the redundancy of miRNAs, there is potential for a small set of miRNAs to be involved in driving and coordinating the expression of these hallmark characteristics, and the miRNAs involved are likely to change during tumour progression as the requirements of cells alter. Thus, understanding the role of various miRNAs at different stages of prostate cancer progression, along with their expression changes during those stages, may inform the search for prognostic biomarkers and even yield potential therapeutic targets. Specifically in prostate cancer, the regulation of miRNAs by the androgen receptor signalling pathway and their influence on this pathway is also of interest. The interaction of miRNAs with other key signalling pathways in prostate cancer such as PTEN/AKT is also discussed.

Whilst much of the complexity of miRNA involvement in cancer development both in general, and specifically in prostate cancer, remains to be fully elucidated, the importance of miRNA in several key oncogenic pathways in prostate cancer has been demonstrated [8].

### MiRNAs and avoidance of apoptosis

There appear to be a number of miRNAs that interact with the apoptotic pathway in prostate cells, and their dysregulation may promote apoptosis avoidance in prostate cancer via a number of mechanisms, chief among them being regulation of the p53 tumour suppressor gene and E2F1-3 transcription factor genes.

The tumour suppressor gene p53 stimulates apoptosis via upregulation of the Noxa and Puma proapoptotic proteins [7]. MiR-125b targets p53-dependent apoptosis by interfering with p53 and its target genes p21 and Puma. MiR-125b can also stimulate p-53 independent apoptosis in p-53 deficient cells via the protein product of the ink4a/ARF locus, p14(ARF) [9]. This suggests a potential role in prostate cancer progression, and indeed enforced expression of miR-125b has been shown to induce prostate tumour growth in both intact and castrate mice [10]. The miR-15-16 cluster influences cell survival by inhibiting translation of the Bcl2 oncoprotein. Bcl2 exerts anti-apoptotic pressure by inhibiting Noxa and Puma. Downregulation of these miRNAs has been demonstrated in 80% of prostate cancers, and results in increased Bcl2 activity, favouring cell survival [11]. Loss of miR-15-16 has additional effects on cell proliferation by upregulation of cyclin D1, which facilitates G1/S phase transition. The loss of this miRNA cluster can also activate the procarcinogenic Wnt pathway [12]. The importance of miR-15-16 in prostate tumour growth has been further emphasised by the observation that reconstitution of these miRNAs may lead to regression of prostate cancer xenografts in mice [12]. MiR-21 has been shown to target programmed cell death 4 (PDCD4) [13] and the phosphatase and tensin homologue (PTEN) [14] both of which stimulate apoptosis via the p53 pathway, resulting in inhibition of these apoptotic triggers.

The E2F1-3 transcription factors play a key role in the regulation of the cell cycle and apoptosis [15]. It has been shown that E2F1-3 bind to promoter sites for the miR-17-92 cluster and consequently upregulate transcription of that group of miRNAs. This group of miRNAs, especially miR-20a, in turn downregulate expression of the E2F1-3 group [16], thereby producing a negative-feedback loop that maintains E2F1-3 expression levels at a relatively constant level. In prostate cancer cells however, miR-17-92 expression may be upregulated, resulting in E2F1-3 suppression and consequently apoptosis avoidance [16]. MiR-20a expression levels are progressively higher in benign, low-grade malignant and high-grade malignant prostate tissue respectively, perhaps suggesting a role in tumour progression [17]. Other miRNAs have also been shown to downregulate E2F1-3 expression including miR-25 [18] and miR-205 [19].

MiRNA expression has also been demonstrated to control other pro-apoptotic genes. Fas Associated Factor 1 (FAF1), a component of the death-inducing signalling complex, is frequently downregulated or lost in a number of human tumour types [20]. FAF1 has been shown to be down-regulated by miR-24, resulting in a reduction in apoptosis in DU-145 hormone-resistant prostate cancer cells *in vitro*[21]. The pro-apoptotic protein TNFSF10 by contrast is upregulated by miR-145 [22]. TNFSF10 (also known as TNF-related apoptosis inducing ligand or TRAIL) is part of the tumour necrosis factor super-family, and contributes to innate anti-tumour immunosurveillance [23].

### MiRNA and cell invasion and migration

In addition to enhanced cell survival and proliferation, carcinogenesis also requires cells to develop the ability to invade the basement membrane and metastasise to distant sites [7]. To achieve this, the cell must become detached and motile and avoid anoikis (a form of apoptosis that occurs when a cell become detached from its substratum) [24]. Granulin has been demonstrated to facilitate cell motility and avoidance of anoikis in a number of tumour types as well as having other procarcinogenic effects such as enhancing epithelial cell division [25]. In prostate cancer, granulin has been shown to facilitate migration and anchorage-independent growth in both androgen-dependent and independent cell lines, although the effect was more marked in androgen-independent cells [26]. Granulin expression is regulated by members of the miR-15 - miR-107 group, and miR-107 in particular has been shown to downregulate granulin expression in prostate cancer cells *in vitro*[27].

Downregulation of miR-145 has also been suggested to lead to enhanced cell proliferation, migration and invasion in prostate cancer. Fuse and colleagues demonstrated that the capacity of PC3 and DU145 prostate cancer cells to proliferate, migrate, and invade was impaired by transfection with miR-145 [28]. Their study demonstrated down-regulation of Fascin homolog 1 (FSCN1) by miR-145, which may be the mechanism by which this occurs.

MiR-143 is also downregulated in prostate cancer, and has been shown *in vitro* to inhibit proliferation and migration of prostate cancer cells by suppressing V-Ki-ras2 Kirsten rat sarcoma viral oncogene homolog (KRAS) expression, thereby inhibiting the EGFR/RAS/mitogen-activated protein kinase (MAPK) pathway [29]. This also resulted in an increase in cell sensitivity to docetaxel. MiR-133 [30] and miR-146a [31] have also been shown to target EGFR and to produce similar anti-proliferative, anti-migratory effects in androgen-insensitive prostate cancer cell lines *in vitro.*

Epithelial-mesenchymal transition (EMT) is a developmental regulatory programme whereby an epithelial cell undergoes a number of alterations that allow it to assume a mesenchymal cell phenotype. These include loss of normal polarity and adhesion, enhanced migratory and invasive capability, resistance to apoptosis and increased production of extracellular matrix [32]. EMT plays a physiological role in embryonal development and wound healing, but is also implicated as one of the principal ways in which carcinoma cells can acquire attributes that allow invasion and metastasis [7].

MiR-205 is downregulated in prostate cancer cells, and this has been implicated in the stimulation of EMT. Prostate cancer cells transfected with miR-205 demonstrated enhanced expression of epithelial adhesion molecules such as E-cadherin, and displayed other features of a more epithelial phenotype, suggesting that miR-205 downregulation may be partly responsible for stimulating EMT [19]. The authors identified a number of targets of miR-205 including N-chimaerin, ErbB3, E2F1, E2F5, ZEB2, and protein kinase Cϵ, and identified the latest of these as being particularly important in regulating EMT. In subsequent work, the role of miR-205 in promoting basement membrane deposition, by limiting the abundance of the ΔNP63α protein, was also clarified. Interestingly, miR-205 does this by attenuating ΔNP63α proteasomal degradation rather than via the more typical miR/mRNA interaction [33].

MiR-143 and miR-145 have been identified as potential regulators of EMT [34]. Both are downregulated in bony metastases arising from prostate cancer, and transfection into PC3 cells has been shown to lead to a more epithelial phenotype *in vitro*, with increased E-cadherin and reduced fibronectin expression [34]. The same group demonstrated that cells overexpressing miR-143 and miR-145 had a reduced propensity to bony invasion *in vivo* in a murine intra-tibial injection model [35], and that in a cohort of 22 patients, reduced expression levels of miR-143 and miR-145 correlated with higher Gleason grade, PSA, and bony metastasis [34].

Further work has suggested that miR-29b may also play a tumour suppressive role by inhibiting EMT [36]. MiR-29b expression levels were reduced in prostate cancer tissue compared to patient-matched non-tumour tissue, and investigators found increased E-cadherin expression and reduced N-Cadherin, Twist, and Snail expression in cells transfected with miR-29b, suggesting that miR-29b inhibits EMT.

### MiRNAs and androgen signalling

There appears to be a complex interaction between androgen signalling in prostate cancer, microRNA expression, and various key pathways in prostate cancer development. In essence, certain miRNAs have been shown to be regulated by androgen-receptor (AR) mediated signalling whilst others are involved in modulating the function of the AR signalling pathway.

### AR-regulated miRNAs

MiR-125b has an androgen-responsive element within the promoter region of its gene [10]. Mi-R125b upregulation has been demonstrated to result in androgen-independent growth in prostate tumour xenografts in castrate mice [10] perhaps through its anti-apoptotic effects (see MiRNAs and avoidance of apoptosis).

### MiR-21

*In vitro*, transfection of LnCaP cells with a miR-21 expressing retrovirus has been shown not only to stimulate androgen-dependent cell growth, but also to rescue cells from androgen-deficient growth arrest [37]. This indicates that miR-21 may also mediate castrate-resistant prostate cancer (CRPC) development. Li and colleagues [38] recently established that tissue miR-21 expression levels may have clinical importance. They evaluated miR-21 expression levels in a cohort of 169 radical prostatectomy tissue samples and found increased miR-21 expression was associated with pathological stage, lymph node status, extracapsular extension and biochemical recurrence. They went on to demonstrate *in vivo* tumour growth repression in a mouse model treated with a miR-21 inhibitor.

MiR-32 is a further example of an androgen-regulated miRNA that is upregulated in CRPC. MiR-32 upregulation is associated with downregulation of the protein BTG2, and reduced BTG2 staining in radical prostatectomy specimens has been shown to predict tumour progression [39].

### MiR-27a

Androgen-stimulated upregulation of miR-27a results in reduced prohibitin expression [40]. Prohibitin is a known tumour-suppressor gene and co-repressor of the androgen receptor, and miR-27a mediated downregulation of its protein product results in increased expression of androgen receptor target genes and increased prostate cancer cell growth.

### MiR-141

A study by Waltering *et al*. attempted to profile differential miRNA expression in androgen treated and untreated LnCaP cells *in vitro* and in prostate cancer xenografts in intact and castrated mice. MiR-141 demonstrated the greatest androgen-dependent expression in that study [41]. MiR-141 is overexpressed in various forms of human epithelial malignancy including prostate cancer [42]. Separate analysis of prostate epithelial and stromal cells has demonstrated miR-141 expression to be epithelium-restricted [6].

#### MiRNAs influencing androgen signalling

Whilst some miRNAs are expressed in an androgen-dependent manner, others appear to modulate various aspects of androgen signalling in prostate cancer. MiR-221 and miR-222 overexpression have been demonstrated to attenuate androgen-induced growth in LnCaP cells *in vitro*, and to promote androgen-independent growth [43]. MiR-221 down-regulation has demonstrated clinical significance in a study that found progressively reduced expression levels in benign tissue, primary prostate cancer, and lymph node metastatic tissue respectively [44]. Reduced miR-221 levels were shown to be linked to increasing Gleason score and recurrence rate in a large (n = 92) cohort of high-risk prostate cancer patients in the same study.

Other miRNAs that modulate the androgen pathway include miR-331-3p and Mir- let7c. Down-regulation of miR-331-3p has been demonstrated to result in an increase in ERBB-2 expression, a tyrosine-kinase receptor thought to play a role in progression from androgen-dependent to independent growth in some prostate cancers [45]. Mir let-7c has been shown have tumour suppressive potential by attenuating androgen receptor expression, through targeting its c-Myc-driven transcription [46].

### MiRNAs and the PTEN/AKT pathway

The PTEN/AKT pathway plays an important role in the development of prostate cancer, and especially in the progression to castrate-resistant disease [47]. Enhanced AKT signalling results in cell proliferation and growth, enhanced survival, cell-cycle progression and angiogenesis [48]. PTEN negatively regulates AKT, and therefore acts as a tumour suppressor. PTEN loss commonly occurs via somatic genetic changes, but miRNAs can also downregulate PTEN. Tian and colleagues identified four miRNAs (miR-19b, miR-23b, miR-26a and miR-92a) that downregulate PTEN expression and enhance cell proliferation *in vitro*[49]. Similar findings were noted for miR-153 by Wu and colleagues [50]. Poliseno and colleagues demonstrated that the miR-106b-25 cluster can also downregulate PTEN in prostate cancer cells. Interestingly, this cluster lies within an intronic region of the minichromosome maintenance complex component 7 (MCM7) gene, which the authors found to be overexpressed in prostate cancer and to co-operate with the miR106b-25 cluster in tumour development [51]. The same group identified another fascinating aspect of non-coding RNA behaviour by discovering the role that the PTENP1 pseudogene plays in PTEN regulation. Like all pseudogenes, the mRNA transcript of PTENP1 is not translated into a functional protein, but by possessing similar miRNA binding sites to PTEN, it competitively binds miRNAs that would otherwise bind to and downregulate the PTEN mRNA product. Loss of PTENP1 therefore enhances miRNA-mediated downregulation of PTEN, promoting tumour development [52].

Wang and colleagues identified that loss of the zinc finger and BTB domain-containing protein 7a (Zbtb7a) gene accelerates oncogenesis in PTEN loss-driven prostate cancers via a SRY box 9 (SOX9) dependent pathway for cellular senescence bypass and invasion [53]. Both PTEN and Zbtb7a gene transcripts are targeted by the mir106b-25 cluster, providing further evidence of the importance of miRNA interaction with the PTEN pathway in prostate cancer development.

### MiRNAs and TMPRSS2:ETS gene fusions

TMPRSS2:ETS gene fusions are present in around 50% of prostate cancers in surgical case series [53]. A chromosomal rearrangement results in the fusion of the androgen-sensitive promoter of the transmembrane protease serine 2 gene (TMPRSS2) gene with one of the ETS family of transcription factors (most commonly the v-ets erythroblastosis virus E26 oncogene homolog gene (ERG)) [54]. This results in androgen-dependent overexpression of ERG, which has a number of downstream effects, including the induction of EMT [55]. Kim and colleagues found that this occurs via ERG-dependent downregulation of miR-200c expression, and that miR-200c transfection could inhibit ERG-induced EMT *in vitro*[56]. The epidermal growth factor (EGF)/Src tyrosine kinase pathway also contributes to the induction of EMT in TMPRSS2:ERG positive prostate cancers, and Kao and colleagues demonstrated that this interaction may be mediated by downregulation of miR-30, which normally suppresses ERG expression [57].

Gordanpour *et al.* identified that miR-221 downregulation is also a feature of TMPRSS:ERG fusion-positive prostate cancer [58]. MiR-221 downregulation has been shown favour androgen-independent tumour growth and is associated with a more aggressive prostate cancer phenotype [44].

### MiRNAs and EZH2-dependent DNA methylation changes

The enhancer of zeste homologue 2 (EZH2) gene is overexpressed in prostate cancer [59] and interacts with DNA methyl transferases to influence DNA methylation [60]. EZH2 is a target of ERG, and therefore overexpressed in TMPRSS2:ERG-positive prostate cancers [61]. Borno and co-workers however, also identified a miRNA-dependent mechanism for EZH2 overexpression in TMPRSS2:ERG negative tumours [62]. This occurs via hypermethylation of the miR-26a locus, resulting in its downregulation. The group found that loss of miR-26a led to upregulation of EZH2 in TMPRSS2:ERG negative tumours. Further work by Cao and colleagues has also identified miR-101 as a downregulator of EZH2 [63].

### Circulating miRNAs in prostate cancer

Understanding of the functional role of miRNA dysregulation in prostate cancer development, and identification of miRNA up- and down-regulation in prostate cancer tissue has led to attempts to determine whether circulating miRNA levels in serum or plasma samples could be used as biomarkers to aid prostate cancer diagnosis, or to improve prediction of tumour behaviour. Indeed, the potential of circulating miRNAs as serum biomarkers has been explored in a number of tumour types [64].

Mitchell *et al.* [65] established the presence of mature miRNAs in a stable form in human plasma, by analysing the 18-24 nucleotide fraction of plasma RNA from a healthy donor. In the same study, the authors established the principle of secretion of tumour-associated miRNAs into the circulation by analysing the sera of prostate cancer xenograft bearing mice. They demonstrated an increase in two miRNAs (miR-629 and -660) that they had identified as being upregulated in the prostate cancer cells prior to implantation, but no increase in other non-cancer associated miRNAs. They then went on to compare the sera of 25 patients with metastatic prostate cancer with 25 healthy controls. They chose six candidate miRNAs (miR-100, miR-125b, miR-141, miR-143, miR-205, and miR-296) that had shown differential expression in prostate cancer in previous studies, but which were not identified in a cloning analysis of the sera of healthy volunteers. They found elevated levels of all miRNAs except miR-205, which was not detected at quantifiable levels in either cancer patients or controls. MiR-141 was the most significantly increased, with 46-fold overexpression in cancer vs. control. MiR-141 levels could predict the presence of prostate cancer with 60% sensitivity at 100% specificity, although it should be noted that all prostate cancer patients in this study had metastatic disease.

A further study by Lodes *et al.*[65] analysed sera from patients with a number of malignancies using a micro-array hybridisation technique. They identified 15 miRNAs that were upregulated in the sera of prostate cancer patients vs. healthy controls (miR-16, -92a, -103, -107, -197, -34b, -328, -485-3p, -486-5p, -92b, -574-3p, -636, -640, -766, -885-5p). This study did not confirm the finding of increased miR-141 expression; however, the group of prostate cancer patients was small (n = 6), with several of the patients being pre-treated with chemotherapy. In addition, no qRT-PCR confirmation of the micro-array results was performed.

Moltzahn and colleagues evaluated a group of 36 prostate cancer patients stratified into 3 groups of 12 based on their CAPRA score (a prognostic index for prostate cancer), and compared them with a group of 12 healthy controls [66]. They identified significant inaccuracy in previously published methods by comparing miRNA expression in cells from wild-type, Dgcr8 knockout, and Dicer knockout mice, which lack canonical and all miRNAs respectively. They demonstrated a group of 10 miRNAs that demonstrated differential quantification in a fluid-chip based high throughput analysis and individual qRT-PCR analyses. Mir- 223, -26b, -30c and miR-24 were downregulated in the cancer group and miR-874, -1274a, -1207-5p, -93 and miR-106a were upregulated. Within those 10, they were able to identify some miRNAs whose expression varied significantly between risk groups, e.g. miR-24 steadily decreased with risk, while miR-106a steadily increased with risk.

Agalogu *et al.*[67] analysed serum levels of three miRNAs in 51 patients with prostate cancer (subdivided into localised/locally advanced and metastatic) and 20 healthy controls. They identified increased levels of miR-21 and miR-221 in the prostate cancer group as a whole compared to the controls. miR-141 was not significantly elevated in the group as a whole, but when the metastatic group were considered in isolation, all three miRNAs were significantly elevated, with mir-141 being the most elevated of the three.

Gonzales and colleagues [68] further investigated the role of miR-141 as a biomarker in advanced prostate cancer by looking at a retrospective cohort of 21 patients with metastatic prostate cancer. They analysed miR-141 levels using qRT-PCR along with lactate dehydrogenase (LDH), prostate specific antigen (PSA), and circulating tumour cell count (CTC) in a number of stored blood samples at taken at varying intervals during the patient’s clinical course. Increasing miR-141 levels demonstrated a significant ability to predict clinical progression via univariate regression modelling, with an odds ratio of at least 8. miR-141 levels also correlated with changes in the other biomarkers under study. The authors suggested that miR-141 may therefore be a suitable biomarker for progression in metastatic prostate cancer, but accepted that larger, prospective studies would be required to validate that contention.

Zhang and co-workers [69] analysed serum miR-21 levels in 20 patients with localised prostate cancer, 20 with metastatic prostate cancer who remained androgen-sensitive, 10 with hormone-resistant disease, and 6 controls with BPH only. They demonstrated elevated miR-21 levels in patients with hormone-resistant prostate cancer and in those with androgen-sensitive metastatic disease whose PSA was greater than 4 ng/ml. There was no difference in miR-21 expression between those with localised prostate cancer and the control group. Additionally, they found that amongst the hormone-resistant group, miR-21 levels were higher in those patients who demonstrated no response to cytotoxic treatment with docetaxel.

Brase *et al.*[70] screened sera from a small cohort of patients with metastatic and locally advanced prostate cancer using a micro-array based technique to quantify 667 miRNAs and identified five that were upregulated in the metastatic group (miRNA-375, miRNA-9*, miRNA-141, miRNA-200b and miRNA-516a-3p). They went on to analyse the most promising candidate miRNAs from their screening study (miR-141, miR-375, and miR-200b) in a larger cohort of patients (n = 45), and found that miR-375 and miR-141 levels could distinguish high-risk (Gleason grade ≥ 8 or metastases) from Gleason 7 prostate cancers with greater accuracy than PSA. They went on to perform the same analyses in a larger cohort (n = 71) of patients including a greater number of high-risk/metastatic patients. They demonstrated that both miR-141 and miR-375 levels were predictive of lymph node positive disease in that group, and that miR-141 could distinguish Gleason ≥8 disease from Gleason 7. Additionally, they went on to demonstrate increased tissue expression of these markers in prostate cancer tissue samples compared to benign prostate tissue.

Selth *et al*. used a mouse model of prostate cancer to identify candidate miRNAs that were overexpressed compared to healthy control mice. They identified upregulation of miR-141, miR-298, miR-346 and miR-375 in both the mouse model and the serum of men with castrate-resistant prostate cancer compared to controls [71]. The same group more recently identified three miRs (miR-141, miR-146b-3p and miR-194) that predicted biochemical recurrence in men following surgery for intermediate-risk prostate cancer [72].

Bryant *et al.*[73] sought to specifically evaluate whether circulating miRNAs within microvesicles or exosomes could be used as biomarkers in prostate cancer. They analysed sera from a cohort of 78 prostate cancer patients and 28 normal control individuals, who were men who had PSA levels <10 ng/ml with negative prostate biopsies. They included a filtration step in their protocol, aimed at isolating purely the microvesicle component of the serum, and treated the resulting filtrate with RNase to remove non-vesicle associated RNA. They analysed the resulting samples with a micro-array panel of 742 miRNAs, finding a total of 11 miRNAs to be upregulated in cancer vs. controls (miR-107, -130b, -141, -2110, -301a, -326, -331-3p, -432, -484, -574-3p, and -625) and one to be down-regulated (miR-181a-2). When exclusively non-metastatic prostate cancer patients were compared to controls, a smaller subset of those miRNAs retained their differential expression levels. When metastatic patients were compared to non-metastatic patients, a different group of 16 miRNAs were found to be differentially expressed, including miR-375, which was also identified as a candidate marker of advanced disease by Brase *et al.*[70]. The group went on to confirm the increased circulating levels of miR-107 and miR-574-3p in prostate cancer cases vs. controls using individual qRT-PCR assays, and likewise the differential quantification of miR-221, miR-375, and miR-141 in metastatic cancers vs. non-metastatic. They also demonstrated increased circulating miR-375 and miR-141 levels in a separate cohort of metastatic prostate cancer patients (n = 47) when compared with a group of patients with no disease recurrence following radical prostatectomy (n = 72). In that subsequent cohort, they analysed the miRNA content of larger microvesicles and smaller exosomes separately, and found increased miR-375 and miR-141 levels in both fractions.

A further recent publication by Huang *et al*. using RNA sequencing verified the finding of miR-375 overexpression in circulating microvesicles in castrate-resistant prostate cancer, along with miR-1290 also [74]. The group where able to confirm this finding in a larger cohort (n = 100) of patients, and found expression of these miRs to correlate with survival in this group.

Interestingly, recent work from Lázaro-Ibáñez *et al*. has demonstrated that genomic DNA can also be retrieved from extracellular vesicles in prostate cancer patients, analysis of which could provide a further source of serum biomarkers [75].

Bryant *et al.* in their study also looked at levels of five miRNAs (miR-107, miR-574-3p, miR-375, miR-200b, miR-141) in post digital rectal examination urine samples [73]. They demonstrated that miR-107 and miR-574-3p levels were significantly higher in prostate cancer cases when compared with controls, indicating that this may also be a valid sample type for miRNA analysis.

Nguyen and co-workers [76] undertook genome-wide miRNA profiling of 84 patients with various stages of prostate cancer, and again identified miR-141 and miR-375 upregulation in castrate-resistant patients compared to those with localised disease. MiR-378 was also over-expressed in that group, whilst miR-409-3p was under-expressed.

Chen *et al.*[77] in a more recent study in Chinese patients undertook profiling of serum samples from prostate cancer (n = 25) and a control group (n = 17) with benign hyperplasia only. They identified a further panel of five miRNAs, which they verified as possessing the ability to distinguish between prostate cancer and benign patients in a larger cohort.

Shen and colleagues [78] evaluated four miRNAs in plasma samples from 82 prostate cancer patients and found that a combination of miR-20a, miR-21, miR-145 and miR-221 could distinguish localised prostate cancer of varying risk groups. These last two studies illustrate the potential value of combining several miRNA assays in order to improve diagnostic or prognostic accuracy.

## Conclusions

There is evidence from *in vitro* and *in vivo* studies that alteration in miRNA function plays a role in prostatic carcinogenesis. miRNA dysregulation influences a number of critical cellular processes involved in this process, including but not limited to: stimulation of the cell cycle, avoidance of apoptosis, epithelial-mesenchymal transition and modulation of AR-mediated signalling. Further understanding of the functional importance of miRNA dysregulation may allow the development of novel therapeutic strategies involving miRNA augmentation or inhibition in the future.

Circulating miRNA profiling in prostate cancer patients has been carried out by various investigators, but studies thus far have involved small numbers of patients, and a variety of methodologies, and have yielded heterogeneous results. However, the potential to detect circulating miRNAs in serum and potentially, in urine, clearly exists.

Furthermore, despite the variability of results, there are a number of miRNA targets that have both a demonstrable functional role in prostate cancer pathogenesis and have demonstrated over- or under-expression in serum samples in at least one study (summarised in Table 1). The investigation of these miRNAs, either singularly or as part of a panel, in larger, prospective patient cohorts will help to define their potential role as diagnostic and prognostic biomarkers in the future.

**Table 1:** **Summary of miRNAs with known functional role in prostate cancer development and differential regulation in serum**

MicroRNA	Function	Up/Downregulated in Serum	Relevant disease stage	Findings from existing studies
MiR-20a	Attenuates the pro-apoptotic action of E2F1-3 [16]	Upregulated [73, 78]	Localised and Metastatic	Distinguishes low/intermediate/high risk localised disease in combination with MiR-21, MiR-145, MiR-221 [78]
MiR-21	Inhibits apoptosis by targeting PDCD4 and PTEN [13, 14]. Can stimulate androgen-independent growth [37]	Upregulated [67, 69, 78]	Localised and Castrate-resistant	Improves diagnostic accuracy of PSA when combined with miR-141 [67] Distinguishes low/intermediate/high risk localised disease [78] Predictive of response to Docetaxel in castrate-resistant disease [50].
MiR-107	Granulin production [27]	Upregulated [73]	Localised	Upregulated in cancer vs. benign in serum microvesicles and urine [73]
MiR-125b	Suppresses p14(ARF) to modulate both p-53 dependent and independent apoptosis [9].	Upregulated [6]	Not elucidated	Distinguishes cancer from control [6]
MiR-143	Suppresses KRAS expression, inhibiting the MAPK pathway. Regulates EMT [29]	Upregulated [6]	Not elucidated	Distinguishes cancer from control [6]
Mir-141	Expression is controlled by androgens [41]. Limited to epithelium [6].	Upregulated [65, 67, 68, 73, 79]	Advanced/Metastatic disease	Distinguishes metastatic and localised disease [70, 76] Predicts biochemical recurrence following radical prostatectomy
MiR-145	Inhibits proliferation, migration, and invasion. Downregulates FSCN1. Inhibits EMT [34].	Downregulated [34, 78]	Localised and metastatic	Correlated with higher Gleason grade, PSA, and bony metastasis [34] Distinguishes low/intermediate/high risk localised disease [78]
MiR-221	Favours androgen-dependent growth. Downregulation may be linked to castrate-resistant state [43, 80].	Upregulated [67, 78] (although downregulation in tissue also reported [81]	Localised and metastatic	Distinguishes low/intermediate/high risk localised disease in combination with MiR-21, MiR-145, MiR-221 [54]
MiR-331-3p	Down-regulation increases ERBB-2 expression. Associated with androgen-independent growth [45]	Downregulated [73]	Not elucidated	Distinguishes cancer from control [73]
Mir-375	Stimulates proliferation via downregulation of Sec23a [82]	Upregulated [70, 73, 76]	Localised and metastatic	Distinguishes metastatic and localised disease [70, 73, 76]
